# Experiences of patients and family members with follow-up care, information needs and provider support after identification of Lynch Syndrome

**DOI:** 10.1186/s13053-023-00273-1

**Published:** 2023-12-19

**Authors:** Ryan Mooney, Yelena P. Wu, Kelsey Kehoe, Molly Volkmar, Wendy Kohlmann, Cathryn Koptiuch, Kimberly A Kaphingst

**Affiliations:** 1 Huntsman Cancer Institute, University of Utah, Salt Lake City, UT USA; 2 Department of Dermatology, University of Utah, Salt Lake City, UT USA; 3 Department of Psychology, University of Massachusetts Boston, Boston, MA USA; 4 VA Medical Center, National TeleOncology Service, Durham, NC USA; 5 Department of Communication, Huntsman Cancer Institute, University of Utah, Salt Lake City, UT USA

**Keywords:** Lynch Syndrome, Hereditary Cancer, Patient experiences, Surveillance, Screening adherence

## Abstract

**Background:**

Lynch Syndrome is among the most common hereditary cancer syndromes and requires ongoing cancer surveillance, repeated screenings and potential risk-reducing surgeries. Despite the importance of continued surveillance, there is limited understanding of patient experiences after initial testing and counseling, the barriers or facilitators they experience adhering to recommendations, and how they want to receive information over time.

**Methods:**

A cross-sectional, observational study was conducted among 127 probands and family members who had received genetic testing for Lynch Syndrome. We conducted semi-structured interviews to determine proband and family member experiences after receiving genetic testing results including their surveillance and screening practices, information needs, and interactions with health care providers. Both closed-ended and open-ended data were collected and analyzed.

**Results:**

Both probands (96.9%) and family members (76.8%) received recommendations for follow-up screening and all probands (100%) and most family members (98.2%) who tested positive had completed at least one screening. Facilitators to screening included receiving screening procedure reminders and the ease of making screening and surveillance appointments. Insurance coverage to pay for screenings was a frequent concern especially for those under 50 years of age. Participants commented that their primary care providers were often not knowledgeable about Lynch Syndrome and surveillance recommendations; this presented a hardship in navigating ongoing surveillance and updated information. Participants preferred information from a knowledgeable health care provider or a trusted internet source over social media or support groups.

**Conclusions:**

Probands and family members receiving genetic testing for Lynch Syndrome generally adhered to initial screening and surveillance recommendations. However, factors such as insurance coverage and difficulty finding a knowledgeable healthcare provider presented barriers to receiving recommended follow-up care. There is an opportunity to improve care through better transitions in care, procedures to keep primary care providers informed of surveillance guidelines, and practices so that patients receive reminders and facilitated appointment setting for ongoing screening and surveillance at the time they are due.

## Background

Lynch Syndrome is among the most common hereditary cancer syndromes [1], and is caused by pathogenic germline variants in one of four mismatch repair genes: *MLH1*, *MSH2*, *MSH6*, and *PMS2* [2]. It is estimated that 1 in 279 individuals in the population carry a heterozygous pathogenic variant in one of the DNA mismatch repair genes, conferring a diagnosis of Lynch Syndrome [3]. Receiving the diagnosis of Lynch Syndrome sets in motion a cascade of personal, familial, and medical consequences that continues over a lifetime and affects a family over generations. Lynch Syndrome is passed through families in an autosomal dominant inheritance pattern and is characterized by a predisposition to colorectal cancer as well as endometrial, small bowel, ovarian, urinary tract, stomach, pancreatic, and brain cancers [1]. Probands may learn of their diagnosis after completing a genetic referral for a high-risk cancer; subsequent cascade genetic testing can then be performed for other at-risk family members through family variant testing. The diagnosis of Lynch Syndrome may be unexpected in the case of a proband diagnosis or instead could be precipitated by a family member’s diagnosis and subsequent recommendation for testing.

The maximum benefits of screening for Lynch Syndrome result from following recommended preventive care [4]. Individuals with Lynch Syndrome are recommended to undergo frequent and repeated cancer surveillance activities to minimize cancer-related morbidity and mortality [4]. Based on National Comprehensive Cancer Network (NCCN) guidelines [5], surveillance recommendations may include surgeries, screenings, medications and/or medical appointments. While recommendations vary depending upon the specific gene involved, recommended risk-reducing surgeries may include hysterectomy and bilateral salpingo-oophorectomy. Screening recommendations may include colonoscopy, esophagogastroduodenoscopy, endometrial biopsy, transvaginal ultrasound, urinalysis, dermatological exams, and pancreatic imaging. Additionally, risk reducing medications may be considered including daily aspirin use in regard to risk for colorectal cancer and hormonal contraception in regard to risk for gynecological cancers [5].

Initiation of ongoing surveillance, potential preventative surgeries and continued follow up may take several pathways: through a specialty clinic connected with a cancer center or large academic center or, more commonly, referral back to the individual’s primary care provider (PCP) immediately after diagnosis or after initial surgeries and/or screenings. While life-long care is critical for individuals with Lynch Syndrome, many factors exist that may serve as barriers to successful navigation of follow up care. In addition, as more is learned about effective management strategies for individuals with Lynch Syndrome, management guidelines are continually updated which makes it even more difficult for individuals with Lynch Syndrome to receive the most appropriate care [6]. It remains unclear how successful patients with Lynch Syndrome are in navigating their follow-up care, and research is needed to understand transitions in care between genetic specialists providing the diagnosis and initial management recommendations and the providers expected to oversee the long-term follow-up care [6].

While some resources to assist in long-term management are being created, dissemination of education on a diverse range of topics on Lynch Syndrome is needed [7]. For instance, Schneider et al. [4] reported the follow-up experiences of a small sample of patients with Lynch Syndrome and their PCPs, and found little follow-up communication between genetics providers and patients after diagnosis and posttest counseling in regard to coordinating check in appointments. In addition, there was limited educational support by PCPs who expressed their belief that such follow-up was provided by the genetics specialty. As a result, patients with Lynch Syndrome did not have a resource for ongoing and updated screening or preventive surgery guidelines. In this study, one-third of study participants reported they had taken responsibility for keeping up and tracking their own care [4]. Additionally, in a 16 year follow up study, Mittendorf et al. [8] found that patients were more likely to adhere to colorectal cancer screening recommendations compared to screening recommendations for extracolonic Lynch Syndrome related cancers. Patients and providers from this study participated in interviews and developed suggestions for addressing the Lynch Syndrome related care gaps identified in the study. These suggestions included use of clear documentation of Lynch Syndrome in the medical record, links to Lynch Syndrome surveillance recommendations located in the electronic medical record (EMR), automated reminders for both patients and providers indicating when a surveillance recommendation is overdue, and proactive outreach from medical genetics in regard to updating patient’s screening recommendations [8]. However, prior research of patient experiences after receiving follow-up recommendations is limited. In order to add to this knowledge base, we examined the experiences, surveillance practices, information needs, and interactions with health care providers of individuals with a diagnosis of Lynch Syndrome.

## Methods

We utilized a cross-sectional, observational design and conducted semi-structured interviews to capture proband and family member experiences with receiving screening and surveillance recommendations, facilitators and barriers to completing these recommendations, and ongoing informational needs. The study’s recruitment and study procedures are described in more detail in Petersen et al. [9], which reported separate aims and results related to patterns of family communication at the time of a Lynch Syndrome diagnosis.

### Participant eligibility and recruitment

Participants were recruited through the Huntsman Cancer Institute, a National Cancer Institute designated comprehensive cancer center in Salt Lake City, Utah. Study eligibility included being a member of a family with a confirmed pathogenic Lynch Syndrome variant, being 18 years or older, and able to speak and understand English. All probands carried a known pathogenic variant. Family participants were members of previously-identified families with Lynch Syndrome but could have been tested for the familial variant with a positive result, been tested for the familial variant with a negative result, or had not been tested. Potential participants were notified by letter of their eligibility for the study. Those who did not opt out received two follow-up phone calls. The initial invitation letter was mailed to 298 potential participants, and 127 (43%) were enrolled. Of individuals who did not enroll (171), the study team was unable to contact 143, 6 were deceased, 4 did not meet eligibility criteria, and 18 declined participation. Enrolled participants included probands (n = 32) and their family members (n = 95).

### Procedures

Research staff conducted individual semi-structured interviews with participants by telephone and digitally recorded the interviews. Participants completed the informed consent process at the start of the interview. Proband interviews were on average, 37 min, with the shortest interview lasting 22 min and the longest interview lasting one hour and seven minutes. Family member interviews lasted on average, 33 min, with the shortest interview lasting 14 min and the longest interview lasting one hour and 27 min. Parallel interview protocols with similar questions were implemented with probands and family members [9]. During the interview, the interviewer entered the participant’s response into an online REDCap form designed by the study team. Both closed and open-ended questions were included on the interview protocol. Open-ended questions were designed to generate more detailed responses to interview domains described below in Table 1. A separate coder reviewed 20% of the recorded interviews and independently coded responses to assess inter-coder reliability with the interviewer’s coding. The overall percent agreement for the proband interviews was 92.5%. The overall percent agreement for the family member interviews was 95.8%. Participants received a $20 gift card for completing the interview. The study procedures were reviewed and approved by the Institutional Review Board of the University of Utah.

**Table 1:** Interview Protocol

Domain	Questions
Demographic and clinical characteristics	Age, race, ethnicity, sex, marital status, cancer status, and insurance status. Familial pathogenic variant was determined via medical record.
Discussion of Lynch Syndrome with a provider	● Did you discuss the diagnosis of Lynch Syndrome with a healthcare provider? ▪ If so, who did you discuss this with?
Ongoing management and surveillance	● Based on your test result, what clinical follow-up was recommended for you? ▪ Who made this recommendation? ● **For Probands**: Since being diagnosed with Lynch Syndrome, what cancer screening(s) have you gotten? ● **For Family Members**: Since receiving your test results, what cancer screening(s) have you received? ▪ What made it easier for you to get cancer screening(s)? ▪ What has made it more difficult for you to get cancer screening(s)?
Education and information regarding Lynch syndrome	● Are you satisfied with the amount of information that you have received about Lynch Syndrome? ▪ Why were you satisfied with the amount of information you received? ▪ Why were you not satisfied with the amount of information you received? ● After hearing a list of examples of ways participants might continue to learn about new Lynch syndrome developments. They were asked: ▪ Which of the examples I mentioned would be the most helpful for you? ▪ Which of the examples I mentioned would be the least helpful for you?

### Measures

The interview protocol had four domains that collected information about participant demographic and clinical characteristics, discussion of Lynch Syndrome with provider, ongoing management and surveillance, and education and information regarding Lynch Syndrome. See Table 1.

### Analysis

Descriptive statistics (mean, standard deviation, frequencies) were calculated to summarize demographic characteristics. Responses to closed-ended questions about Lynch Syndrome diagnosis, ongoing management and surveillance, facilitators and barriers to clinical follow-up, and education and information preferences regarding Lynch Syndrome were categorized and described with descriptive statistics. For each interview domain, open-ended responses were coded using content and thematic analysis methodology [10]. The research team met to discuss and define coding categories. The coders then independently applied the coding categories to participants’ responses. After this, the research team met to compare and reconcile discrepancies and to identify themes from the codes. To complete the analysis, we examined data from both the closed-ended and open-ended analyses for each domain of interest (i.e., Lynch Syndrome diagnosis and ongoing management and surveillance; barriers and facilitators to clinical follow-up; education and information regarding Lynch Syndrome).

## Results

### Participant characteristics

Table 2 summarizes participants’ demographic characteristics. The majority of probands and family members identified as female (n = 25; 78.1% and n = 64; 67.4%, respectively), Caucasian (n = 30; 93.8% and n = 90; 94.7%, respectively), and married (n = 25; 78.1% and n = 70; 73.7%, respectively). Most probands were over the age of 40 (n = 28; 87.5%), while about half (n = 51; 53.7%) of family members were over the age of 40. Almost all (n = 29; 90.6%) probands had been diagnosed with cancer, while the minority (n = 22; 23.2%) of family members had received a prior cancer diagnosis. Of the 127 participants, the majority (n = 81; 63.8%) came from a family with a pathogenic mutation in the genes *MLH1* or *MSH2*. Nearly all family member participants (n = 86; 90.5%) had pursued genetic testing for Lynch Syndrome. A majority (n = 56; 59.0%) received a positive test result, (n = 30; 31.6%) received a negative test result, and the remaining (n = 9; 9.5%) had not pursued genetic testing at the time of the interview.

**Table 2:** Participant Characteristics

	Probands		Family Members	
Characteristic	n	%	n	%
Gender				
Male Female	7 25	21.9 78.1	31 64	32.6 67.4
Race/Ethnicity				
Non-Hispanic, Caucasian Other	30 2	93.8 6.3	90 5	94.7 5.3
Age				
<40 years of age ≥40 years of age missing	4 28 0	12.5 87.5 0.0	43 51 1	45.3 53.7 1.1
Cancer status				
Prior cancer No prior cancer	29 3	90.6 9.4	22 73	23.2 76.8
Gene with pathogenic variant in family				
*MLH1* *MSH2* *MSH6* *PMS2*	6 10 10 6	18.8 31.3 31.3 18.8	24 41 9 21	25.3 43.2 9.5 22.1
Personal pathogenic variant status				
Positive Negative Not tested	32 0 0	100.0 0.0 0.0	56 30 9	59.0 31.6 9.5
Marital status				
Married/living as married Widowed/divorced/other Single	25 6 1	78.1 18.1 3.1	70 12 13	73.7 12.6 13.7
Insurance status				
Private Public No insurance	17 15 0	53.1 46.9 0.0	63 29 3	66.3 30.5 3.2

### Lynch syndrome diagnosis and ongoing management and surveillance

The vast majority of probands (n = 32; 96.9%) and family members (n = 73; 76.8%) reported discussing the Lynch Syndrome diagnosis in the family with a healthcare provider. The majority of probands (n = 36; 83.9%) and about half of family members (n = 36; 49.3%) discussed the diagnosis with a specialty physician. About half of the probands (n = 16; 51.6%) discussed their Lynch Syndrome diagnosis during a routine appointment, whereas the majority (n = 45; 64.3%) of family members discussed the diagnosis during an appointment specific to their Lynch Syndrome diagnosis.

All probands (n = 32; 100%) reported having received a recommendation for clinical follow-up, such as cancer screenings or surgical procedures. Almost all (n = 55; 98.2%) family members who tested positive received a recommendation for clinical follow-up. Most (n = 22; 73.3%) of family members who tested negative did not receive a recommendation for clinical follow-up. We found that a specialty physician was the most common type of provider to make clinical follow-up recommendations for both probands and family members (n = 60; 52.6%), followed by genetic counselors (n = 42; 36.8%) and PCPs (n = 6; 5.2%).

Participants reported what screenings they had received since their Lynch Syndrome diagnosis or since receiving their genetic test results. Overall, 96.8% (n = 31) of probands and 87.5% (n = 49) of family members who had a positive test result reported completing one or more recommended screenings (see Table 3). Approximately 4% of family members who tested positive (n = 2) reported that no screenings had been recommended and so they had not taken any action. In addition, 12.5% of family members who tested positive (n = 7) reported having not received any recommended screening despite having received a recommendation for screening. A higher percentage of probands than family members who tested positive for the familial variant reported receiving key screenings, including colonoscopy (n = 28, 87.5%; n = 43, 76.8% respectively). Interestingly, probands were also more likely (n = 6, 19.4%) to report receiving general population screening such as mammograms than family members who tested positive (n = 4, 8.2%). Participants who tested positive were also asked to identify surgical interventions they had received. Some female probands reported having had a hysterectomy (n = 9; 28.1%) or oophorectomy (n = 5; 15.6%), and some female family members who tested positive reported having had a hysterectomy (n = 12; 14.1%) or oophorectomy (n = 8; 9.4%). In addition, two family members (3.6%) and no probands reported having had a colectomy. However, we did not collect information about the reason for the colectomy.

**Table 3:** Screenings Completed by Participants with Lynch Syndrome who Received a Recommendation for Screening

Screening performed	Probands (n = 31)		Family Members (n = 49)	
	n	%	n	%
Colonoscopy	28	90.3	43	87.8
Endoscopy	12	38.7	26	53.1
Mammogram	6	19.4	4	8.2
Skin screening	4	12.9	8	16.3
Urine sample	4	12.9	5	10.2
Endometrial biopsy	1	3.2	4	8.2
Ovarian ultrasound	0	0	4	8.2
Don’t known/Not sure	2	6.5	0	0
*Other	12	38.7	12	24.5

*Other family member: H. pylori testing (n = 1), Pap smear (n = 3), Pancreatic US (n = 1), Fecal test (n = 1), CT scan unspecified (2); MRI unspecified (n = 1).

*Other proband: CEA blood screen (n = 2), Cystoscope (n = 1), MRI unspecified (n = 5), CT scan unspecified (n = 5), Chest X-ray (n = 1).

### Facilitators and barriers to clinical follow-up for lynch syndrome

The most commonly reported facilitators to probands receiving screenings included being sent a reminder (n = 6; 19.4%) and the ease of making an appointment (n = 6; 19.4%). For example, a proband participant stated, “I got a reminder in the mail, plus looking at MyChart that said I’m overdue for colonoscopy.” The most commonly reported facilitators to family members receiving screenings included having insurance coverage (n = 16; 21.3%), having screening appointment scheduling facilitated by clinic staff (n = 10; 13.3%), and the ease of making an appointment (n = 8; 10.7%). For instance, a family member shared, “I had a reminder from my doctor and that made it easy. And they were accommodating and easy to schedule.”

Issues with insurance was the most commonly-reported barrier to receiving screenings for both probands (n = 12; 37.5%) and family members (n = 19; 20.0%). When discussing barriers to receiving screenings in the semi-structured interviews, one proband stated, “You have to fight the insurance company because they say you can’t be screened this often.” A family member also expressed having difficulties with insurance: “I haven’t had [a screening] yet because my insurance doesn’t cover it because I’m under 50. We don’t have money to pay and that was definitely a huge challenge. But I will begin having them when I’m 50 and my insurance begins covering it.” Family members reported other barriers to receiving screenings including the emotional burden, the difficulty of preparing for screening procedures, their doctors, particularly PCPs, not knowing about Lynch Syndrome causing participants to feel that their care was not being prioritized. In the semi-structured interviews one family member stated, “Just medical professionals don’t know much about Lynch [Syndrome] so receiving help is difficult because they don’t treat it as anything urgent.”

One theme that emerged from the semi-structured interviews was the experiences of both probands and family members with PCPs who had limited knowledge about Lynch Syndrome. For example, one proband stated, “I was not able to talk with my primary care physician because he does not really know anything about Lynch Syndrome.” Another proband expressed a desire for more doctors to know about Lynch Syndrome: “I wish more doctors would know about it and be more proactive with patients.” Family members who were interviewed expressed similar experiences with providers who had limited knowledge. One family member said, “Only if the PCP knew about Lynch [Syndrome]. My PCP needed me to tell them about it.” Another family member’s doctor needed to search for Lynch Syndrome online: “And he didn’t know a lot about Lynch Syndrome he had to look it up online.” Another family member decided to go to a specialist instead of their PCP due to the PCP’s lack of knowledge around Lynch Syndrome: “I talked with an oncologist who is very familiar with Lynch Syndrome. I made a colonoscopy appointment. He wasn’t up to date on it but I gave him some information. He is, however, the first doctor that I’ve talked to who knows what Lynch Syndrome is. My regular physician didn’t know anything really up to date on Lynch Syndrome at all. So, I go to this other doctor for that.”

### Education and information regarding lynch syndrome

Probands and family members were asked about their satisfaction with the amount of information they received from their provider about Lynch Syndrome. The majority of probands (n = 28; 87.5%) and family members (n = 70; 81.4%) reported being satisfied with the amount of information they received. When asked why they were satisfied, probands (n = 9; 33.3%) and family members (n = 19; 27.1%) indicated they felt they understood Lynch Syndrome. Many reported doing their own research on Lynch Syndrome (e.g., on the Internet) and knowing where to find the information for which they were looking. One proband stated, “I feel like I understand everything that is going on and what I need to do. I have a good grasp. I’ve talked to numerous doctors and received different perspectives.” Similarly, a family member said, “Yes, [I’m satisfied with the amount of information I’ve received]. I don’t feel like I need to be an expert but I feel like I was properly educated about it. I know what it is and what the risks are of having it and what I would have to do if I had it. It has been good.” Of the probands (n = 4; 12.5%) and family members (n = 16; 18.6%) who indicated they were not satisfied with the amount of information they received about Lynch Syndrome, many reported that there was not enough information available about Lynch Syndrome, they wanted more information, and they were not sure what information was accurate and inaccurate.

Participants were asked about what ways would be the most helpful for them to continue learning about Lynch Syndrome. Probands and family members indicated that the following ways would be the most helpful in learning about Lynch Syndrome in the future: discussing new developments with their PCP as part of an annual check-up (n = 6, 20.7%; n = 26, 30.2%), access to a clinic specializing in caring for people with Lynch Syndrome (n = 6, 20.7%; n = 13, 15.1%), and general websites about Lynch Syndrome to use when they had questions (n = 7, 24.1%; n = 16, 18.6%). One proband who expressed that websites would be the most helpful stated, “I find websites are a good start and you can go from there to other information”. Similarly, one family member stated, “An absolute webpage with everything on there based on Lynch Syndrome [would be the most helpful for my family learning about Lynch Syndrome].” One proband who felt access to a specialty clinic would be most helpful said, “Access to a specialist [would be] very helpful because I’ve educated a lot of my physicians like my family medicine doctor.” One family member expressed that access to a specialty clinic would make them more confident in their care: “I would trust a specialty clinic because I would know they know what they are talking about. It would make me confident in care.”

In the interviews, both probands and family members reported that social media and support groups would not be helpful for them and their families in terms of receiving future information and updates on Lynch Syndrome. One proband stated, “I don’t see [social media] as something I would feel comfortable using for that information.” One family member said, “I wouldn’t go to social media to talk about Lynch Syndrome.” When asked about support groups, one proband said, “Support group, not for our family.” One family member said, “A support group [would be the least helpful]. I feel that I wouldn’t want to sit and talk to people about it, I would rather talk to doctors.”

## Discussion

In this study, we found that the majority of probands and family members reported discussing their Lynch Syndrome diagnosis with a healthcare provider. The vast majority of probands discussed their diagnosis with a specialty physician, whereas just under half of family members did so. All probands and almost all family members who received a positive test result reported receiving a recommendation for clinical follow-up. In terms of who made the recommendations, specialty physicians were the most common type of provider, followed by genetic counselors, for both probands and family members, whereas PCPs were the least common provider to make follow-up recommendations. Most probands and family members who tested positive reported receiving at least one recommended screening. Of family members who had received a screening recommendation, just over 10% reported that they had not yet followed through with their recommendation. The majority of probands and family members with a positive test result reported receiving a colonoscopy, whereas proband and family member reports of receiving other screenings, such as endoscopies, was less common. This finding is consistent with Mittendorf et al. [8], who found high adherence to colonoscopy but varied adherence to other screening recommendations in patients diagnosed with Lynch Syndrome.

Our findings highlighted a number of important barriers to receiving recommended follow-up care. A primary reported barrier for probands and family members to receiving recommended screenings and follow-up care was lack of insurance coverage, which was particularly true for those under 50 years of age. This is consistent with findings from Campbell-Salome et al. [11], which found that cost and issues with insurance made it difficult for patients with Lynch Syndrome to adhere to recommended screenings, such as endoscopies and colonoscopies. While time consuming, healthcare providers may need to advocate for insurance payment for patient screening. An additional barrier reported by family members and probands in our study was experiences with PCPs who had limited knowledge about Lynch Syndrome. This finding indicates a need for patients to be directed to a knowledgeable clinician for further guidance. It also highlights the need for increased education on Lynch Syndrome for providers, including PCPs, who may need to manage clinical surveillance activities. Findings also suggest that there is poor coordination and problems in transitions in care from the diagnosing provider and genetic counselor to the PCP. Better coordination may help PCPs manage continued surveillance and screening. Together, these patient-reported barriers likely make adherence to recommended Lynch Syndrome clinical follow-up more difficult and thus require patient persistence to negotiate and obtain their care.

Patients also shared experiences that made adherence to screening and surveillance appointments easier. Reminders to schedule screening was most commonly cited along with the ease in making appointments. Utilizing advances in electronic medical record capabilities to send patient reminders, allow patients to schedule appointments online, and display screenings due during appointments with PCPs, offers the opportunity to improve continuity in care for high risk patients including those with Lynch Syndrome, so they are supported in adhering to screening recommendations. These clinical decision support features could support a tailored lifelong, gene-specific management plan for people with Lynch Syndrome, which is thought to be crucial in building a patient’s confidence in their treatment plan [12].

### Availability of educational resources

Generally, patients reported that they had received enough information from their provider when receiving their initial Lynch Syndrome diagnosis. Many patients also reported doing research on their own and reported knowing where to go if they have questions. For those who were not satisfied with the amount of information received, this was attributed to a lack of available information or being concerned about accuracy of the source. When asked about what ways would be most helpful for receiving information on new developments regarding Lynch Syndrome, patients reported wanting to discuss new developments with a PCP, having access to a specialty clinic, or having general websites to visit where they could have some of their questions about Lynch Syndrome answered. Although patients reported ideally wanting to talk with their PCP about new developments, many patients also reported that PCPs lack knowledge relating to Lynch Syndrome. This also highlights the need for increased medical education for providers. In addition, there may be value for genetic counseling practices to establish a registry so they can notify those who have received testing when there are updates and new information about Lynch Syndrome genes and screenings recommendations with updates also sent to the patients’ PCPs. Given that many patients are doing research on their own and would like to be able to access general websites about Lynch Syndrome, there is a need for websites or other forms of information with quality and accurate information on Lynch Syndrome. Interestingly, while patients reported that they would like to receive further information on Lynch Syndrome from websites, many reported that receiving information on Lynch Syndrome from social media would be the least helpful due to privacy concerns and concerns regarding false information. This finding is inconsistent with findings from Campbell-Salome et al. [11], who reported that patients turned to social media and blogs to gather information on Lynch Syndrome when they did not receive sufficient information from providers or from online sources to reduce their fears and uncertainty regarding Lynch Syndrome.

### Limitations

While this study provides valuable insights into the experiences with surveillance, medical management and informational needs of patients after genetic testing for Lynch Syndrome, there are some limitations that should be noted. In our study, time between initial Lynch Syndrome diagnosis and study participation varied between participants, which could impact accuracy of recall and time to develop habits with screening and surveillance. Additionally, screening data was self-reported and therefore was not verified from the medical record. This study was performed at a large academic cancer center in which some participants had access to genetics specialty care follow up that might not be generalizable to most patients seeking screening and surveillance. In addition, our population was primarily white and female, and results may not be generalizable to other populations.

### Future directions

Future research in this area could focus on examining screening and medical management practices in patients with Lynch Syndrome over a longer period of time using prospective cohort methods or using longitudinal health system data. Our study identified the need to develop and test improved workflows for transitions in care as well as methods to disseminate updated screening, surveillance and medical management guidelines to patients, family members and primary care providers. It may be important to explore how patients and their providers keep up to date and adapt to changes in screening and management guidelines. In terms of clinical improvements, those providing diagnostic, genetic testing and counseling services and ongoing management should explore aids to improving timely adherence to surveillance guidelines such as the use of automated systems in the electronic medical record that are designed to track patient screening frequency and alert providers when a referral for next screening should be placed. This system could come with gene specific information and recommendations that are put together by genetics professionals and kept up to date.

## Conclusion

Probands with Lynch Syndrome and affected family members will require ongoing screening and management to reduce morbidity and mortality associated with a number of different cancers. Our study highlights barriers to adherence with ongoing screening for this population. While patients with Lynch Syndrome were mostly adherent to their first round of screenings, systemic level factors posed significant barriers to ongoing screening adherence. These included inadequate health insurance coverage including policies that do not recognize screening cadence based on high risk status and the difficuty for patients in finding knowledgeable primary care clinicians for continued survelliance. Additionally, participants reported wanting to learn about new developments with Lynch Syndrome management as screening guidelines evolve but they were not confident that their general health care providers had access to updated information. Findings from this study highlight the need for improved work flows, better dissemination of guideline updates and more effective handoffs in care. Health policy work is also needed so that insurance coverage reflects the surveillance and screening guidelines for those with Lynch Syndrome and their affected family members.

## Data Availability

The datasets used and/or analyzed during the current study are available from the corresponding author on reasonable request and subject to institutional agreements for data sharing.

## Abbreviations

NCCN: National Comprehensive Cancer Network
PCP: Primary care provider
EMR: Electronic Medical record

## References

[1] Giardiello FM, Allen JI, Axilbund JE, Boland CR, Burke CA, Burt RW, Church JM, Dominitz JA, Johnson DA, Kaltenbach T, Levin TR (2014). Guidelines on genetic evaluation and management of Lynch syndrome: a consensus statement by the US Multi-society Task Force on Colorectal cancer. Gastroenterology.

[2] Kohlmann W, Gruber SB, Lynch S. 2004 Feb 5 [updated 2018 Apr 12]. In: Adam MP, Ardinger HH, Pagon RA, Wallace SE, Bean LJH, Mirzaa G, Amemiya A, editors. GeneReviews® [Internet]. Seattle (WA): University of Washington, Seattle. 2019.

[3] Win AK, Jenkins MA, Dowty JG, Antoniou AC, Lee A, Giles GG, Buchanan DD, Clendenning M, Rosty C, Ahnen DJ, Thibodeau SN (2017). Prevalence and penetrance of major genes and polygenes for Colorectal cancer. Epidemiol Biomarkers Prev.

[4] Schneider JL, Goddard KA, Muessig KR, Davis JV, Rope AF, Hunter JE, Peterson SK, Acheson LS, Syngal S, Wiesner GL, Reiss JA (2018). Patient and provider perspectives on adherence to and care coordination of lynch syndrome surveillance recommendations: findings from qualitative interviews. Hereditary Cancer in Clinical Practice.

[5] NCCN. Genetic/Familial High-Risk Assessment: Colorectal, Version 1.2023. 2023.

[6] Menahem B, Alves A, Regimbeau JM, Sabbagh C (2019). Lynch syndrome: current management in 2019. J Visc Surg.

[7] Corines MJ, Hamilton JG, Glogowski E, Anrig CA, Goldberg R, Niehaus K, Salo-Mullen E, Harlan M, Sheehan MR, Trottier M, Ahsraf A (2017). Educational and psychosocial support needs in Lynch syndrome: implementation and assessment of an educational workshop and support group. J Genet Couns.

[8] Mittendorf KF, Hunter JE, Schneider JL, Shuster E, Rope AF, Zepp J, Gilmore MJ, Muessig KR, Davis JV, Kauffman TL, Bergen KM (2019). Recommended care and care adherence following a diagnosis of Lynch syndrome: a mixed-methods study. Hereditary Cancer in Clinical Practice.

[9] Petersen J, Koptiuch C, Wu YP, Mooney R, Elrick A, Szczotka K, Keener M, Pappas L, Kanth P, Soisson A, Kohlmann W (2018). Patterns of family communication and preferred resources for sharing information among families with a Lynch syndrome diagnosis. Patient Educ Couns.

[10] Patton MQ. Qualitative research & evaluation methods: integrating theory and practice. Sage publications; 2014 Oct. p. 29.

[11] Campbell-Salome G, Buchanan AH, Hallquist ML, Rahm AK, Rocha H, Sturm AC (2021). Uncertainty management for individuals with Lynch Syndrome: identifying and responding to healthcare barriers. Patient Educ Couns.

[12] Edwards P, Monahan KJ (2022). Diagnosis and management of Lynch syndrome. Frontline Gastroenterol.

